# Missense mutation in DISC1 C-terminal coiled-coil has GSK3β signaling and sex-dependent behavioral effects in mice

**DOI:** 10.1038/srep18748

**Published:** 2016-01-05

**Authors:** James Dachtler, Christina Elliott, R. John Rodgers, George S. Baillie, Steven J. Clapcote

**Affiliations:** 1 School of Biomedical Sciences, University of Leeds, Leeds LS2 9JT, UK; 2 Institute of Cardiovascular and Medical Sciences, University of Glasgow, Glasgow G12 8QQ, UK; 3 Institute of Psychological Sciences, University of Leeds, Leeds LS2 9JT, UK

## Abstract

Disrupted-in-Schizophrenia 1 (*DISC1*) is a risk factor for schizophrenia and affective disorders. The full-length DISC1 protein consists of an N-terminal ‘head’ domain and a C-terminal tail domain that contains several predicted coiled-coils, structural motifs involved in protein-protein interactions. To probe the *in vivo* effects of missense mutation of DISC1’s C-terminal tail, we tested mice carrying mutation D453G within a predicted α-helical coiled-coil region. We report that, relative to wild-type littermates, female DISC1^D453G^ mice exhibited novelty-induced hyperlocomotion, an anxiogenic profile in the elevated plus-maze and open field tests, and reduced social exploration of unfamiliar mice. Male DISC1^D453G^ mice displayed a deficit in passive avoidance, while neither males nor females exhibited any impairment in startle reactivity or prepulse inhibition. Whole brain homogenates showed normal levels of DISC1 protein, but decreased binding of DISC1 to GSK3β, decreased phospho-inhibition of GSK3β at serine 9, and decreased levels of β-catenin in DISC1^D453G^ mice of either sex. Interrupted GSK3β signaling may thus be part of the mechanism underlying the behavioral phenotype associated with D453G, in common with the previously described N-terminal domain mutations Q31L and L100P in mice, and the schizophrenia risk-conferring variant R264Q in humans.

Schizophrenia is a severe psychiatric condition characterized by three clusters of symptoms: positive symptoms (psychosis and thought disorder), negative symptoms (social and emotional deficits), and cognitive symptoms^1^,^2^. It is well established from family, twin and adoption studies that genetic factors play an important role in the risk of developing schizophrenia^3^. Disrupted-in-Schizophrenia-1 (*DISC1*) is a genetic susceptibility locus for schizophrenia and related psychiatric disorders^4^. The gene was first identified in a large Scottish family in which a balanced chromosomal translocation t(1;11) disrupting the DISC1 gene in the middle of its ORF co-segregates with schizophrenia and affective disorders^5^,^6^. The t(1;11) translocation is believed to be unique, but subsequent studies have supported *DISC1*’s candidacy as a susceptibility gene for these conditions.

Numerous common and rare *DISC1* missense variants have been associated with the increased risk of psychiatric illness, altered brain morphology or cognitive deficits^7^,^8^. For instance, the major S704 allele of the common S704C variant (rs821616) is associated with increased risk of schizophrenia^9^ and increased severity of positive symptoms at the onset of psychosis^10^, while the minor 264Q allele of the common R264Q variant (rs3738401) is associated with increased risk of treatment-refractory schizophrenia^11^. Case-control mutation studies of *DISC1* have identified rare missense mutations that confer an estimated attributable risk of about 2% in schizophrenia^12^ and 0.5% in bipolar disorder^13^, including R418H that was found in both disorders. Other studies have reported an increased burden of rare missense mutations in a Swedish schizophrenia cohort^14^, and an excess of exon 11 rare missense mutations in schizoaffective disorder^15^. Understanding how relatively subtle changes in the composition of the DISC1 protein may confer behavioral abnormalities is thus important in further elucidating the role of *DISC1* in schizophrenia and related mental disorders.

The full-length DISC1 protein (854 amino acids) is predicted to consist of an N-terminal globular ‘head’ domain (residues 1–350) encoded by exons 1–2 and an α-helical coiled-coil-containing C-terminal tail domain (residues 351–854), encoded by exons 3–13^16^. The DISC1 protein acts as a scaffold, binding over 200 interacting molecules – the so-called ‘DISC1 interactome’^17^ – including PDE4B (phosphodiesterase 4B)^18^, GSK3β (glycogen synthase kinase 3 beta)^19^ and dopamine receptor D_2_^20^. DISC1 carries out multiple functions in the nervous system by interacting with various proteins in different cell compartments from embryonic development until adulthood^16^. Several risk-conferring missense variants in DISC1 are located within known binding sites^16^, so missense variation in DISC1 has the potential to affect a variety of neural processes via disturbed interactions with critical binding partners. Indeed, the major S704 allele of S704C (rs821616) has shown decreased binding to NDEL1 (nuclear distribution gene E-like homolog 1)^21^ but increased binding to NDE1 (nuclear distribution gene E homolog 1)^22^ compared with the minor 704C allele; while the minor 264Q allele of R264Q (rs3738401) has shown decreased binding to GSK3β, increased Y216 autophosphorylation-induced activation of GSK3β, and decreased levels of the GSK3β-specific substrate β-catenin compared with the major R264 allele^23^, thus lending support to a putative pathogenic role.

The linked hypotheses that different DISC1 variants will predispose to different phenotypes and that the phenotype of any given DISC1 variant will be modulated by components within, upstream and downstream of the DISC1 scaffold complex are amenable to experimental testing. Two ethylnitrosourea (ENU)-derived mutant *Disc1* mouse lines have previously been described, each with a different missense mutation in the N-terminal head domain of DISC1: Q31L and L100P^24^. Both mutants have inhibited cortical neuronal proliferation, aberrant neuronal migration, reduced dendritic spine density^25^, reduced brain volumes and deficits in spatial working memory^24^ compared with wild-type mice. Furthermore, the L100P mutants show behaviors, including profound prepulse inhibition (PPI) and latent inhibition (LI) deficits, akin to those seen in some with schizophrenia, which could be reversed by treatment with antipsychotic medications^24^. By contrast, the Q31L mice exhibit a more depressive-like behavior, with abnormalities in social behavior and the forced swim test, partially ameliorated by the antidepressant bupropion, but not by rolipram, a PDE4 inhibitor^24^.

Neither Q31 nor L100 is conserved between mouse and human – corresponding to R35 and S100, respectively, in human DISC1 – so their direct involvement in human conditions is unclear. However, given their potential for structural disruption and proximity to functional domains and protein binding regions, Q31L and L100P may still provide important mechanistic insights into the possible effects of DISC1 variants in humans. Mechanistically, both mutations reduce the interaction of DISC1 with PDE4B, but only Q31L mutant mice showed a decrease in PDE4B activity, consistent with the resistance of Q31L mutants and the contrasting sensitivity of L100P mutants and wild-type mice to treatment with rolipram^24^.

Mutations Q31L and L100P both reduce the interaction of DISC1 with GSK3β^26^,^27^, and via distinct routes lead to increased GSK3β activity^26^,^27^,^28^. L100P mice have decreased AKT-dependent phospho-inhibition of GSK3β at S9 in conjunction with increased DISC1 binding to dopamine receptor D_2_ (DRD2) in striatum^28^, but Y216 autophosphorylation-induced activation of GSK3β was unaltered in both striatum and hippocampus^27^. The same phenomenon was subsequently observed in postmortem striatum samples from schizophrenia patients of unknown *DISC1* genotype^28^. Administration of either an interfering peptide that disrupts DISC1-DRD2 binding or the DRD2 antagonist haloperidol to L100P mice blocked the increase in DISC1-DRD2 complex formation and the decrease in phospho-inhibition of GSK3β, and successfully reversed their schizophrenia-related behavioral deficits^28^. By contrast, Q31L mice have shown increased Y216 autophosphorylation-induced activation of GSK3β in hippocampus, with no changes in phospho-inhibition of GSK3β at S9^26^. The GSK3β inhibitor TDZD-8 ameliorated the behavioral deficits of both Q31L^26^ and L100P^27^ mutant lines, indicating that increased GSK3β activity is part of the mechanism underlying the pathophysiology of their abnormal behavior.

Given the insights provided by the Q31L and L100P mutant lines, and the scope for expanding the *Disc1* allelic series to further refine genotype-phenotype correlations and elucidate DISC1 function in the mouse, we extended the ENU mutagenesis screen to a different part of the *Disc1* locus. Herein, we report the initial analysis of the behavioral effects of a novel ENU-derived mutant *Disc1* mouse line, which has a missense mutation (D453G) in a predicted α-helical coiled-coil region within the C-terminal tail domain of DISC1. D453G occurs within a known GSK3β binding region on DISC1 (residues 356–595)^19^, in which mutation R418H has been found in schizophrenia and bipolar disorder patients^12^,^13^. We report that DISC1^D453G^ mutant mice show altered GSK3β signaling in the brain and sex-dependent behavioral deficits.

## Results

### Identification of missense mutation in DISC1 C-terminal tail

**Figure 1:**
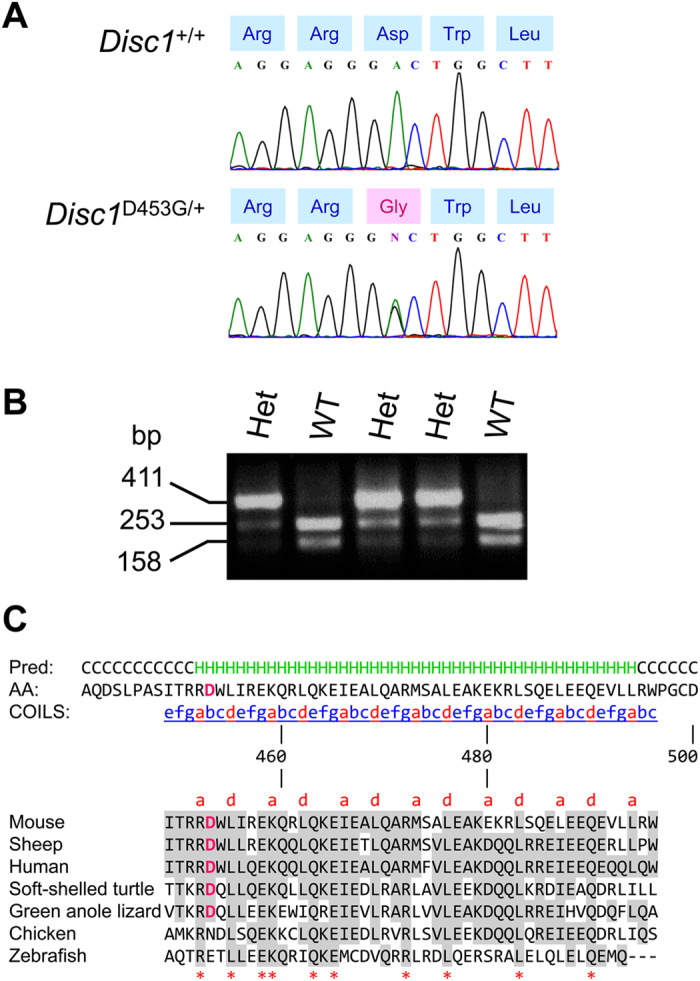
D453G mutation in mouse DISC1. (**A**) DNA sequence chromatogram showing point mutation in *Disc1* exon 5. Transition A1358G converts codon 453 from GAC aspartic acid (Asp) to GGC glycine (Gly). (**B**) *Bse*NI restriction site (5′-ACTGGN*-3′) abolished by the A1358G mutation in *Disc1*. In wild-type (WT) mice, a 411-bp PCR amplicon is cleaved by *Bse*NI into 253-bp and 158-bp fragments. (**C**) Top: PsiPred-predicted secondary structure and COILS server predicted coiled-coil within residues 441–500 of DISC1 are shown and colored accordingly: predicted helices: ‘H’ and colored green above the sequence; coiled-coil heptad repeats: ‘*abcdefg*’, and positions ‘*a*’ and ‘*d*’ shown in red text. Bottom: Alignment of predicted coiled-coil in mouse (*Mus musculus*), sheep (*Ovis aries*), human (*Homo sapiens*), soft-shelled turtle (*Pelodiscus sinensis*), green anole lizard (*Anolis carolinensis*), chicken (*Gallus gallus*) and zebrafish (*Danio rerio*), showing conservation of amino acid D453. Dashes indicate alignment gaps. Residues identical between human and the other species have a gray background, with highly conserved residues indicated by asterisks. D453 is shown in magenta text.

The C-terminal tail domain of mouse full-length DISC1 (NP_777279; 852 residues) spans amino acid residues 351–852 and is encoded by *Disc1* exons 3–13^16^. We screened exon 5 (121 bp; residues 423–464) of *Disc1* (ENSMUSG00000043051) in 7,776 ENU-mutagenized mice in the MRC Harwell ENU DNA archive. In a single mouse (EMRCB/26.2e), we detected an adenine to guanine (A1358G) transition, corresponding to an Asp^453^ (GAC) → Gly (GGC) (D453G) exchange (Fig. 1A), which abolished a *Bse*NI restriction site (Fig. 1B). The exon 5 sequences of the BALB/cAnN and C3H/HeH parental strains are identical (data not shown), indicating that the DISC1^D453G^ mutation arose as a result of ENU administration. We propose that the new allele be designated *Disc1*^*enu1H*^. The aspartic acid at position 453 occurs within a predicted α-helical coiled-coil region, and is conserved across mammals and reptiles, but not birds or fish (Fig. 1C). We used three programs, PolyPhen-2^29^, PMut^30^ and PROVEAN^31^, to predict the potential effect of D453G on the function and structure of the DISC1 protein. PolyPhen-2 predicted D453G to be ‘probably damaging’; PMut predicted D453G to be ‘pathological’, with a reliability value of 7 out of 9; and PROVEAN predicted D453G to be ‘deleterious’. Based on all three programs predicting D453G to affect DISC1 protein function, we bred homozygous mutant and wild-type littermates for assessment in a series of behavioral tests to evaluate the effects of D453G on different domains of the mouse behavioral repertoire.

### Female DISC1^D453G^ mice show hyperlocomotion in the open field test

**Figure 2:**
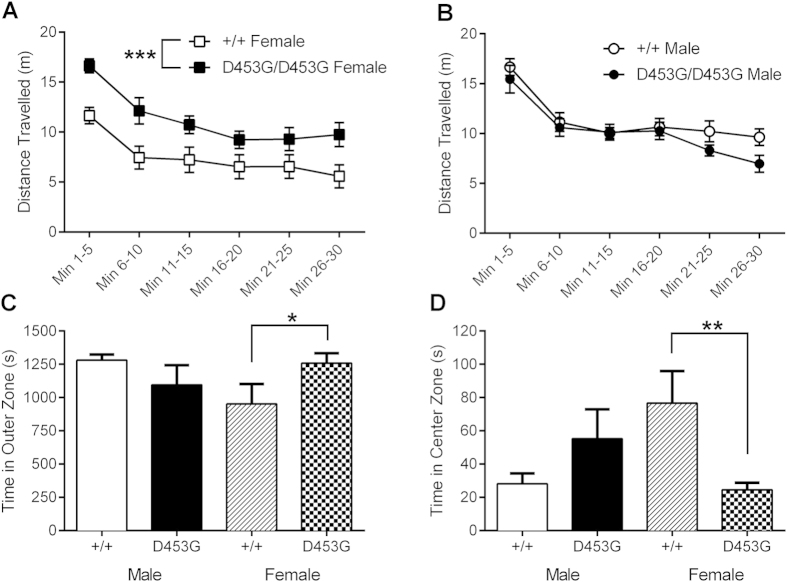
Female DISC1^D453G^ mice show hyperlocomotion and thigmotaxis in the open field. Wild-type (+/+; n = 17, 10 male and 7 female) and DISC1^D453G^ (D453G/D453G; n = 17, 7 male and 10 female) mice were allowed 30 min for free exploration of the arena. (**A**) Female DISC1^D453G^ mice consistently travelled further than female wild-type littermates over the 30 min test period, showing a significant genotypic difference in total distance travelled. (**B**) The distance travelled was not significantly different between male wild-type and DISC1^D453G^ mice. When the arena was broken into outer, intermediate and center zones, female DISC1^D453G^ mice spent significantly more time in the outer zone (**C**), and significantly less time in the center zone (**D**). There were no genotypic differences among males. * P < 0.05, **P < 0.01, ***P < 0.001 vs. female +/+.

Hyperlocomotion in a novel open field is thought to be analogous to psychomotor agitation^32^, a positive symptom of schizophrenia, and has previously been shown by DISC1^L100P^ mice^24^. Throughout the 30 min open field test, female DISC1^D453G^ mice consistently travelled further than female wild-type littermates (Fig. 2A), while there was no obvious difference between male DISC1^D453G^ mice and their wild-type counterparts (Fig. 2B). A repeated-measures three-way ANOVA found that, while there was no main effect of sex (F_(1, 30)_ = 3.66, P > 0.05) or genotype (F_(1, 30)_ = 3.15, P > 0.05), there was significant genotype x sex interaction (F_(1, 30)_ = 10.52, P = 0.003). No other interactions were significant (F < 1). Following up on the significant genotype x sex interaction, a test of simple main effects (SME) revealed a significant difference between female DISC1^D453G^ and wild-type mice (F_(1, 30)_ = 12.59, P = 0.001) but not between male mutants and wild-types (F_(1, 30)_ = 1.08, P > 0.05). Total distance travelled during the first min in the open field was not significantly different between genotypes or sexes (Supplementary Fig. S1; two-way ANOVA: genotype (F(1,30) = 3.37, P = 0.076), sex (F(1,30) < 1), genotype x sex (F(1,30) < 1)).

The natural tendency of mice when first introduced into the open field is to remain close to the walls, and avoid the potentially dangerous open area. This behavior, termed “thigmotaxis”, has been used as an index of anxiety in mice, an interpretation supported by the fact that anxiolytic drugs decrease thigmotaxis^33^. To assess whether the female-specific difference in novelty-induced locomotion could reflect more subtle phenotypes such as anxiety, ambulation in the open field was divided into three different zones (outer, intermediate and center). Female DISC1^D453G^ mice spent significantly more time in the outer zone compared with female wild-types (Fig. 2C; two-way ANOVA: significant genotype x sex interaction (F_(1, 30)_ = 5.87, P = 0.022); SME revealed a significant difference between females (F_(1, 30)_ = 4.56, P = 0.041) but not males (F_(1, 30)_ = 1.67, P > 0.05)). Correspondingly, female DISC1^D453G^ mice spent significantly less time in the center zone than female wild-types, with no genotypic differences among males (Fig. 2D; two-way ANOVA: significant genotype x sex interaction (F_(1, 30)_ = 11.50, P = 0.002); SME reveals a significant different between females (F_(1, 30)_ = 9.96, P = 0.004) but not males (F_(1, 30)_ = 2.6, P > 0.05)). There was an almost significant difference between female mutant and wild-type mice for the time spent in the intermediate zone (Supplementary Fig. S1; two-way ANOVA: significant genotype x sex interaction (F_(1, 30)_ = 4.95, P = 0.034); SME revealed an approaching significant difference between females (F_(1, 30)_ = 3.76, P = 0.062) but not males (F_(1, 30)_ = 1.46, P = 0.24)). The DISC1^D453G^ mutation thus results in female-specific hyperlocomotion (as measured by distance travelled), thigmotaxis and reduced time spent in the center zone, a profile strongly suggestive of an anxiogenic phenotype.

As a final measure of exploration, time spent rearing in the open field was examined. In contrast to the above female DISC1^D453G^ differences, male DISC1^D453G^ mice spent significantly less time rearing, with no genotypic differences between female mice (Supplementary Fig. S1; two-way ANOVA: significant genotype x sex interaction (F_(1, 30)_ = 5.43, p = 0.027); SME revealed a significant difference between males (F_(1, 30)_ = 5.11, p = 0.031) but not females (F_(1, 30)_ = 1.07, p = 0.31)). DISC1^L100P^ mice have previously shown increased grooming^34^, but we found no significant differences for time spent grooming for either DISC1 genotype or sex (Supplementary Fig. S1; two-way ANOVA: all P > 0.05).

### Female DISC1^D453G^ mice exhibit a profile of anxiety but not depression

**Figure 3:**
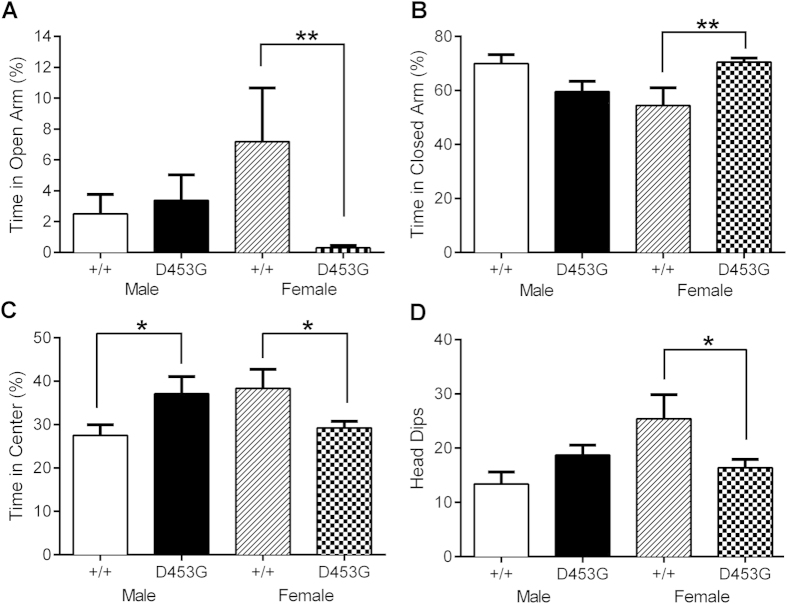
Female DISC1^D453G^ mice exhibit an anxiogenic profile in the elevated plus-maze. (**A**) Female DISC1^D453G^ mice (D453G; n = 10) spent significantly less time than female wild-type littermates (+/+; n = 7) exploring the open arms. There were no genotypic differences among males (P > 0.05). (**B**) Female DISC1^D453G^ mice spent significantly more time in the closed arms than female wild-types, whereas male DISC1^D453G^ mice showed a non-significant trend for spending less time in the closed arms (P = 0.063). (**C**) The center of the elevated plus-maze is where the open and close arms intersect. Male DISC1^D453G^ mice spent significantly more time at the center compared with wild-type males. Female DISC1^D453G^ mice spent significantly less time at the center compared with wild-type females. (**D**) Head dips made on the open arms are indicative of exploratory behavior. Female DISC1^D453G^ mice made significantly fewer head dips compared with wild-type females. *P < 0.05, **P < 0.01 versus +/+ of the same sex.

To further examine whether DISC1^D453G^ results in an anxiety phenotype, as suggested by increased thigmotaxis and reduced center time in the open field, we measured the time spent exploring the open arms of an elevated plus-maze. DISC1^D453G^ females spent significantly less time in the open arms than wild-type females, but there were no genotypic differences in open arm time among males (Fig. 3A; two-way ANOVA: significant genotype x sex interaction (F_(1, 30)_ = 4.83, P = 0.036); SME revealed a significant difference between females (F_(1, 30)_ = 7.61, P = 0.01) but not males (F_(1, 30)_ < 1)). DISC1^D453G^ females remained in the closed arms for significantly longer than wild-type females, whereas DISC1^D453G^ males showed a non-significant trend for spending less time in the closed arms than wild-type males (Fig. 3B; two-way ANOVA: significant genotype x sex interaction (F_(1, 30)_ = 11.98, P = 0.002); SME revealed a significant genotypic difference between females (F_(1, 30)_ = 8.78, P = 0.006) but not males (F_(1, 30)_ = 3.73, P = 0.063)). DISC1^D453G^ males spent more time in the center square than wild-type males, but there were no genotypic differences in center time among females (Supplementary Fig. S2; two-way ANOVA: significant genotype x sex interaction (F_(1, 30)_ = 9.68, P = 0.004); SME revealed a significant difference between females (F_(1, 30)_ = 4.63, P = 0.04) and males (F_(1, 30)_ = 5.06, P = 0.032)). Total distances travelled during the first min and throughout the 5 min elevated plus-maze test were not significantly different between genotypes or sexes (Supplementary Fig. S2, two-way ANOVA: all F_(1, 30)_ < 1).

As a final measure of elevated plus-maze exploration, head dips (downward movement of mouse’s head toward the floor from the open arms) were counted^35^. Female DISC1^D453G^ mice made significantly fewer head dips than female wild-types (Supplementary Fig. S2; two-way ANOVA: significant genotype x sex interaction (F_(1, 30)_ = 7.89, P = 0.009); SME revealed a significant difference between females (F_(1, 30)_ = 6.26, P = 0.018) but not males (F_(1, 30)_ = 2.17, P = 0.15)).

The forced swim and tail suspension tests are established paradigms for the identification of depressive-like behaviors in rodents^36^, in which DISC1^Q31L^ mice have previously shown increased immobility^24^. In the forced swim test, in which time spent floating within an inescapable cylinder of water is measured, there were no significant differences for either genotype or sex (Supplementary Fig. S2, two-way ANOVA, all factors P > 0.05). Similarly, there were no differences for either genotype or sex for the time spent immobile in the tail suspension test (Supplementary Fig. S2, two-way ANOVA, all factors P > 0.05). Taken together, the DISC1^D453G^ mutation appears to result in an anxiogenic profile in females only, without an overtly depressive-like phenotype in either males or females.

### Female DISC1^D453G^ mice show reduced social interaction

Schizophrenia is associated with reduced sociability^37^, and DISC1^Q31L^ mice have previously shown reduced social interaction^24^. To assess the sociability of DISC1^D453G^ mice, we utilized the three-chambered social approach test^38^, which measures the preference of the mouse to either explore a novel adult male conspecific enclosed in a ventilated container (Stranger 1) versus an identical but otherwise empty container.

**Figure 4:**
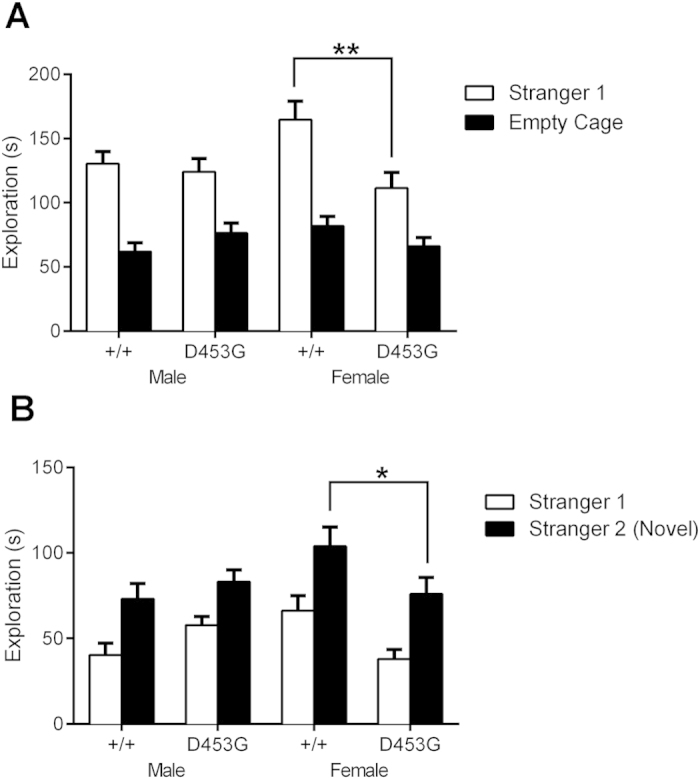
Female DISC1^D453G^ mice show less social interaction. (**A**) Time spent by wild-type (+/+; n = 16, 9 male and 7 female) and DISC1^D453G^ (D453G; n = 17, 7 male and 10 female) mice exploring a novel mouse or an empty container. All groups were able to discriminate between a novel mouse (Stranger 1) and an empty container, but female DISC1^D453G^ mice spent significantly less time than wild-type females interacting with the novel mouse. There was no genotypic difference in time in contact with the novel mouse among males (P > 0.05). (**B**) The mice were then tasked with discriminating between the mouse previously explored and a second novel adult male mouse (Stranger 2). Again, both genotypes and sexes were able to correctly discriminate the second novel mouse, but female DISC1^D453G^ mice spent significantly less time interacting with the novel male mouse. There were no male genotypic differences (P > 0.05). *P < 0.05, **P < 0.01 vs. female +/+.

Notably, female wild-type mice spent significantly longer exploring the novel mouse compared to the empty container than any other group, including female DISC1^D453G^ mice, although all groups showed a preference for exploring the novel mouse (Fig. 4A; RM three-way ANOVA, genotype x sex interaction (F_(1, 29)_ = 7.73, P = 0.009); SME revealed a significant difference between female mice (F_(1, 29)_ = 12.68, P = 0.001) but not male mice (F_(1, 29)_ < 1)). The reduced social interaction of DISC1^D453G^ females compared with wild-type females was not due to deficient ambulation within the arena, as there were no genotypic differences for distance travelled during the test (Supplementary Fig. S3; two-way ANOVA, all P > 0.05).

To examine social recognition, the test mouse then encountered a second novel adult male mouse in the previously empty container (Stranger 2), as well as the now familiar conspecific. Once again, female DISC1^D453G^ mice exhibited lower contact with the novel mouse and the previously introduced mouse than female wild-type littermates (Fig. 4B; RM three-way ANOVA, genotype x sex interaction (F_(1, 29)_ = 9.86, P = 0.004); SME revealed a significant difference between female mice (F_(1, 29)_ = 9.09, P = 0.005) but not male mice (F_(1, 29)_ = 2.08, P = 0.16)), although they could clearly discriminate between Stranger 1 and Stranger 2. Again, this difference cannot be explained by reduced exploration of the arena, as there were no genotypic differences in the total distance travelled (Supplementary Fig. S3; two-way ANOVA, all P > 0.05). Taken together, the DISC1^D453G^ mutation does not appear to disrupt the ability to discriminate between the two unfamiliar mice, a deficiency previously shown by DISC1^Q31L^ mice^24^, but it does reduce total social interaction time in a female-specific manner.

Disruption of nesting behavior has been observed in a genetic mouse model of schizophrenia^39^ and offered as a murine measure of the negative symptom of self-neglect^40^,^41^. We found that nest-building ability was intact in DISC1^D453G^ mice; both genotypes and sexes were able to make nests of similar quality (Supplementary Fig. S3; two-way ANOVA; no main effects or interactions (all F_(1, 30)_ < 1)).

### Male DISC1^D453G^ mice show a deficit in passive avoidance

**Figure 5:**
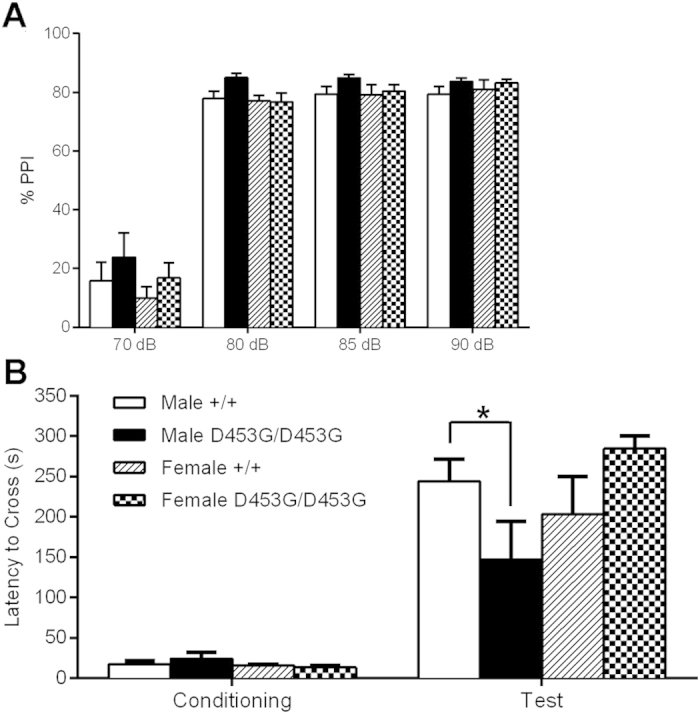
DISC1^D453G^ affects long-term memory of male mice in the passive avoidance test but has no effect on PPI. (**A**) The percentage of PPI at four different tones was measured. DISC1^D453G^ mice (D453G/D453G; n = 17, 7 male and 10 female) showed similar PPI to wild-types (+/+; n = 17, 10 male and 7 female), with no significant differences (P > 0.05). (**B**) Long-term (24 hr) memory was assayed using the passive avoidance test, whereby the mouse must learn to suppress its innate preference for the dark chamber through footshock conditioning. Initially, mice readily cross to the dark chamber (left side, Conditioning). 24 hr later, the latency to cross was measured again (right side, Test). Compared with wild-type mice, DISC1^D453G^ mice more readily crossed to the chamber in which the footshock was delivered. There were no genotypic differences among females (P > 0.05). *P < 0.05 vs. male +/+.

Sensory gating and information processing are frequently disrupted in schizophrenia, and are thought to reflect the cognitive impairments associated with the disorder^42^. Prepulse inhibition, whereby a preceding brief low-intensity stimulus decreases the response to a startle-eliciting stimulus, is disrupted in schizophrenia patients^17^ and in DISC1^L100P^ mice^24^. We found that the DISC1^D453G^ mutation did not alter the acoustic startle response for sound intensities ranging from 70 to 120 dB (Supplementary Fig. S4; three-way ANOVA, all main effects (F_(1, 30)_ < 1), genotype x sex (F_(1, 30)_ = 1.14, P > 0.05), dB intensity x genotype x sex (F_(1, 30)_ = 1.14, P > 0.05)). There were no significant differences in PPI at the differing prepulse intensities between the groups (Fig. 5A; three-way ANOVA, main effects of genotype (F_(1, 30)_ = 2.68, P > 0.05) and sex (F_(1, 30)_ = 1.46, P > 0.05), genotype x sex (F_(1, 30)_ < 1), dB intensity x genotype x sex (F_(1, 30)_ < 1)).

To assess long-term memory, we employed the passive avoidance test, which requires the mouse, via a single footshock conditioning trial, to subsequently inhibit its natural tendency to move from an illuminated chamber to a dark chamber (photophobia). Impairment in passive avoidance has previously been shown by a mouse model of psychosis induced by ketamine^43^,^44^. During the conditioning trial, DISC1^D453G^ and wild-type mice of either sex showed similar latencies to cross to the dark chamber. During the probe trial 24 hours later, male DISC1^D453G^ mice crossed to the dark chamber more readily than male wild-type mice. However, there were no significant genotypic differences between female mice (Fig. 5B; repeated-measures three-way ANOVA; genotype x sex interaction (F_(1, 30)_ = 5.88, P = 0.022); SME revealed no significant genotypic differences between males (F_(1, 30)_ = 1.27, P > 0.05) or females (F_(1, 30)_ < 1) during conditioning, but there was a significant difference between male mice (F_(1, 30)_ = 4.16, P = 0.050) but not female mice (F_(1, 30)_ = 2.96, P = 0.096) during the test 24 hours post-conditioning).

### DISC1^D453G^ mutation disrupts GSK3β signalling

**Figure 6:**
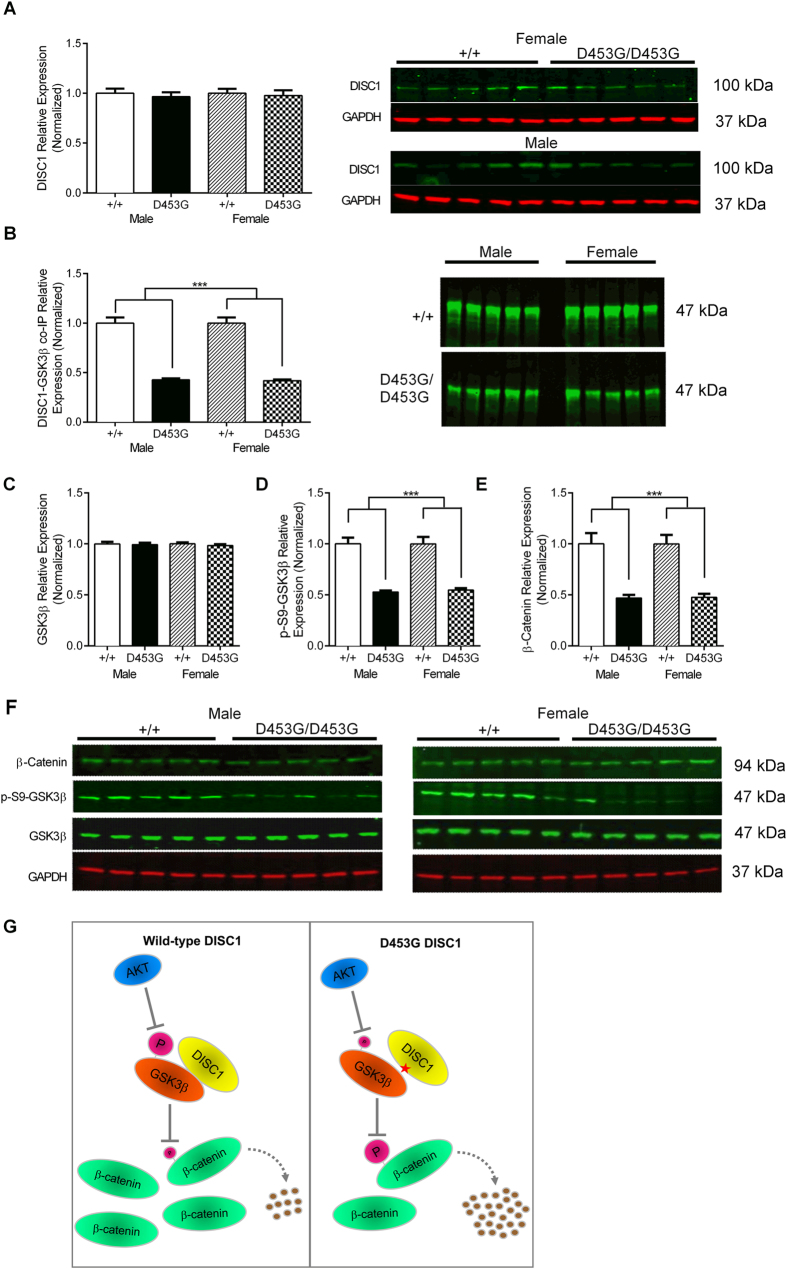
DISC1^D453G^ disrupts GSK3β signaling. (**A**) Western blotting revealed comparable levels of DISC1 protein in whole brain homogenates from wild-type (+/+; n = 10, 5 male and 5 female) and DISC1^D453G^ (D453G/D453G; n = 10, 5 male and 5 female) mice (left side; mean of three gels). Representative blots of DISC1 and GAPDH loading control for male and female DISC1^D453G^ mice (right side). (**B**) The ability of DISC1 to complex with GSK3β was tested by co-immunoprecipitation (co-IP). DISC1^D453G^ significantly reduced DISC1-GSK3β binding in both male and female DISC1^D453G^ mice. (**C**) GSK3β protein levels were comparable between wild-type and DISC1^D453G^ mice of either sex. (**D**) Levels of S9 phosphorylated GSK3β (p-S9-GSK3β) normalized to total GSK3β were significantly decreased in whole brain homogenates from both male and female DISC1^D453G^ mice. (**E**) β-catenin protein levels were decreased in whole brain homogenates from DISC1^D453G^ mice of either sex. (**F**) Representative blots for male (left side) and female (right side) wild-type and DISC1^D453G^ mice. (**G**) Schematic model depicting the effects of D453G on GSK3β signaling. Mutation D453G reduces the interaction of DISC1 with GSK3β, and AKT-dependent phospho-inhibition of GSK3β at S9 is decreased. The less inhibited GSK3β phosphorylates more β-catenin, leading to more ubiquitin-mediated degradation of β-catenin, and reducing the cellular level of β-catenin. Red star: D453G mutation; T-shaped arrows: inhibition; magenta circles: phosphate group, size indicating degree of phospho-inhibition; curved broken arrow: ubiquitin-mediated degradation; brown circles: products of β-catenin proteolysis.

To determine whether the D453G mutation affects expression of the DISC1 protein, whole brain homogenates were subjected to Western blot analysis. No differences were found in the abundance of DISC1 for either genotype or sex (Fig. 6A; two-way ANOVA: no main effects or interactions (all F_(1, 60)_ < 1)). Previous studies have revealed DISC1’s involvement in regulation of the β-catenin/GSK3β pathway, whereby DISC1 inhibits GSK3β activity through direct physical interaction, which reduces β-catenin phosphorylation and stabilizes β-catenin^19^,^23^. However, the GSK3β binding sites on DISC1 have been localized with only limited precision: to a 220-residue region (1–220) at the N-terminus and a 240-residue region (356–595) that encompasses D453 on the C-terminal tail^19^. Missense variants within the N-terminal domain of DISC1 in mice (Q31L and L100P)^26^,^27^ and humans (R264Q)^23^ have been shown to reduce the interaction of DISC1 with GSK3β. To test whether the interaction of DISC1 with GSK3β is altered by the D453G mutation, DISC1-GSK3β binding was assessed in whole brain homogenates by co-immunoprecipitation. In DISC1^D453G^ mice of either sex, DISC1-GSK3β binding was reduced by 57–58% compared with wild-type levels (Fig. 6B; two-way ANOVA: genotype (F_(1, 60)_ = 184.5, P < 0.0001), sex (F_(1, 60)_ < 1, P > 0.05), sex x genotype (F_(1, 60)_ < 1, P > 0.05)). Such a marked reduction in DISC1-GSK3β binding identifies D453 as critical for this interaction.

Decreased S9 phospho-inhibition of GSK3β has previously been observed in striatum from L100P mice^28^, and in striatum and frontal cortex samples from schizophrenia patients of unknown *DISC1* genotype^28^,^45^. We found that the abundance of GSK3β in whole brain homogenates was unaffected by the D453G mutation, with males and females from both genotypes having comparable GSK3β protein levels (Fig. 6C; two-way ANOVA: no main effects or interactions (all F_(1, 60)_ < 1)). However, levels of S9 phosphorylated GSK3β (p-S9-GSK3β), measured as the ratio of p-S9 density to total GSK3β density, were significantly decreased in whole brain homogenates from DISC1^D453G^ mice, compared with wild-type (Fig. 6D; two-way ANOVA: genotype (F_(1, 60)_ = 95.6, P < 0.0001), sex (F_(1, 60)_ < 1, P > 0.05), sex x genotype (F_(1, 60)_ < 1, P > 0.05)). Accumulation of β-catenin has been widely employed as an indirect assay of GSK3β activity on the basis that GSK3β phosphorylates β-catenin, thus targeting it for ubiquitin-mediated degradation by the proteosome, whereas inhibition of GSK3β by lithium causes accumulation of β-catenin in a dose-dependent manner^46^. The decrease in phospho-inhibition of GSK3β in DISC1^D453G^ mice raised the possibility that the overall levels of β-catenin may be decreased. We found that whole brain homogenates from DISC1^D453G^ mice indeed had significantly lower levels of β-catenin (Fig. 6E; two-way ANOVA: genotype (F_(1, 60)_ = 52.0, P < 0.0001), sex (F_(1, 60)_ < 1, P > 0.05), sex x genotype (F_(1, 60)_ < 1, P > 0.05)), consistent with their reduced phospho-inhibition of GSK3β. Thus, the D453G mutation reduces β-catenin abundance (Fig. 6G).

## Discussion

We have conducted an initial analysis of the behavioral effects of a novel missense mutation (D453G) within the C-terminal tail domain of DISC1 in mice with a predominantly C57BL/6N genetic background. The substitution of an aspartic acid (D; polar, hydrophilic, negative charge) by a glycine (G; aliphatic, neutral) at position 453 was predicted to have a harmful effect on the structure and function of DISC1 by all three bioinformatics algorithms utilized, consistent with D453 being conserved across mammals and reptiles. This conservation allows direct comparison across species, but the corresponding residue in human DISC1 (D456) is not the location of a known human disease-associated variant.

In the absence of a published crystal structure for DISC1, we used bioinformatics resources to predict the effects of D453G on DISC1 structure. The COILS program^47^ predicted a 48-residue coiled-coil region corresponding to residues 449-496 in mouse DISC1, encompassing D453. The PsiPred^48^ secondary structure prediction of α-helices corresponding to residues 452–494 overlaid appropriately with the region of coiled-coil helix propensity. This prediction closely matches a region of coiled-coil potential corresponding to residues 452–499 in human DISC1^16^. The hallmark of most coiled-coils is a regular seven-residue sequence pattern known as a heptad repeat, which is often labeled ‘*abcdefg*’, where positions ‘*a*’ and ‘*d*’ are internalized, stabilizing the structure when two or more helices wind around each other to form ‘supercoils’, while the remaining positions are solvent exposed and usually provide surfaces for protein-protein interaction^47^. This is probably true in the case of D453, as it corresponds to position ‘*b*’ in the heptad repeat, and was found by co-immunoprecipitation in the present study to be critical for the interaction of DISC1 with GSK3β in whole brain homogenates. Among the seven vertebrate species examined for DISC1 protein conservation (Fig. 1C), none has a glycine (G) anywhere within the amino acid sequence of the predicted coiled-coil region corresponding to residues 449–496 in mouse DISC1, suggesting that D453G may indeed have a structural impact.

Among the known DISC1 binding partners, we focused specifically on GSK3β because D453G occurs within a known GSK3β binding region on DISC1 (residues 356–595)^19^. Moreover, mutations Q31L and L100P within another GSK3β binding region on DISC1 (residues 1–220) were both previously shown to reduce the interaction of DISC1 with GSK3β^26^,^27^ in the mouse brain. In the present study, we found that whole brain homogenates from DISC1^D453G^ mice showed unaltered levels of DISC1 protein, but decreased binding of DISC1 to GSK3β, decreased S9 phospho-inhibition of GSK3β, and decreased levels of the GSK3β-specific substrate β-catenin (Fig. 6G).

Similarly reduced phospho-S9-GSK3β has previously been observed in striatum from L100P mice^28^, in striatum and frontal cortex samples from schizophrenia patients^28^,^45^, and in peripheral lymphocytes from schizophrenia patients^45^ and drug-free bipolar disorder patients^49^. Human lymphoblast cell lines endogenously expressing the schizophrenia risk variant R264Q have shown decreased levels of β-catenin compared with the major R264 allele^23^. β-catenin is known to regulate the self-renewal of neural progenitor cells and neuronal differentiation in the developing neocortical ventricular zone, subcortical areas of the telencephalon, and ventral midbrain^50^. Hence, interruption of GSK3β signaling could impair neurodevelopment, thus lending support to a putative pathogenic role in neurodevelopmental disorders.

In behavioral tests, we found that neither male nor female DISC1^D453G^ mice exhibited impairment in startle reactivity or prepulse inhibition (Table 1). Female DISC1^D453G^ mice did, however, exhibit novelty-induced hyperlocomotion in the open field, as well as an anxiogenic profile in the open field and the elevated plus-maze, and reduced social exploration of unfamiliar mice in the social approach test. The open arms of the elevated plus-maze were illuminated with an intensity of 200 lux, which is similar to the light levels used in elevated plus-maze experiments that detected an anxiolytic phenotype in *Nos1* knockout mice^51^ and an anxiogenic phenotype in *Alox5* knockout mice^52^. Another anxiety-related behavior that is sometimes measured in the elevated plus-maze is the frequency or duration of freezing (suppression of all movement except that required for respiration)^35^, but the overhead camera that we used with the elevated plus-maze was not of sufficiently high resolution to score freezing accurately.

Male DISC1^D453G^ mice appeared less anxious than DISC1^D453G^ females. However, their decreased rearing in the open field and increased center time in the elevated plus-maze would be more suggestive of an increased anxiety state compared with wild-type males. Although these mice displayed a non-significant tendency to increased center time in the open field, which would normally be interpreted as mild anxiolysis, it should be noted that both the elevated plus-maze and open field tests commenced with the placement of mice at the center of the apparatus. The observed increase in center time in both novel environments may therefore have reflected an initial reluctance by the mice to move from their start position. However, this hypothesis was not supported by an analysis of total distance travelled in the first minute of each test, which failed to reveal any significant main effects or interactions. Male DISC1^D453G^ mice also displayed a deficit in passive avoidance, a behavioral paradigm that reflects the cognitive ability of mice to associate the dark compartment with the aversive event (footshock) experienced in that compartment during training.

Consistent with the decreased inhibitory phosphorylation of GSK3β and the passive avoidance deficit exhibited by DISC1^D453G^ males, mice with postnatal neuronal expression of the phosphorylation defective mutant GSK3β*S9A have also shown impaired long-term memory in passive avoidance^53^. However, the disruption of GSK3β signaling by DISC1^D453G^ was observed in both sexes, so cannot readily account for the sex-dependent behavioral effects of the mutation. Assessing the behavioral effects of the GSK3β inhibitor TDZD-8 in DISC1^D453G^ males and females would help to evaluate the contribution of alterations in GSK3β signaling to their respective behavioral phenotype, as done previously with the Q31L and L100P mutant lines^26^,^27^.

It is theoretically possible that disruption of GSK3β signaling could have different effects in males and females, as the female sex hormone and neuroprotectant estrogen regulates the stability of β-catenin, induces the translocation of β-catenin to the cell nucleus, and regulates β-catenin-mediated transcription in neurons^54^. However, as the current study has no data directly relevant to estrogen’s regulation of β-catenin, further study is needed to empirically test this hypothesis. Since D453 (human D456) occurs within a multi-protein interaction site^16^ and a self-association domain (residues 403–504)^55^, GSK3β is unlikely to be the only interaction partner affected by D453G. By virtue of the complexity of DISC1 interaction with proteins intimately involved in either neurodevelopment or neuromodulatory signaling^17^, other protein interactions and a variety of neural processes could be altered by D453G; their modulation by mechanisms for sex-dependent differences in gene expression^56^,^57^ could contribute to the sexually-dimorphic behavioral phenotype we observed.

Female DISC1^D453G^ mice displayed an anxiogenic phenotype in the elevated plus-maze and open field, whereas males were less affected in these tests. It remains unclear why only female DISC1^D453G^ mice exhibited increased anxiety. In human genetic studies, a single nucleotide polymorphism (SNP) in *DISC1* intron 9 (rs821577) has shown female-specific associations with anxiety, depression and neuroticism in elderly Scottish subjects^58^, and with schizophrenia in a Japanese population^59^, while another *DISC1* intron 9 SNP (rs2295959) has shown female-specific association with schizophrenia in Han Chinese subjects^60^. These findings suggest that variance in DISC1 has stronger effects in females than in males, which could potentially contribute to the sex differences found in the schizophrenia phenotype, such as age at onset, premorbid personality, clinical subtypes, psychosocial function, and responses to therapy^61^.

Transgenic mice with inducible expression of truncated human DISC1 (residues 1–597) under the control of the forebrain-specific *Camk2a* promoter also show sex-dependent behavioral abnormalities^62^. Male transgenics displayed enhanced spontaneous locomotor activity and alterations in social interaction, whereas female transgenics demonstrated deficient reference spatial memory in the Morris water maze^62^. In another mouse study, *Disc1* knockdown specifically in adult-born dentate gyrus neurons resulted in increased mTOR signaling and several behavioral deficits, including enhanced anxiety in the elevated plus-maze, whereas *Disc1* knockdown specifically in hippocampal CA1 neurons did not elicit any behavioral changes^63^. Treatment with rapamycin, an FDA-approved compound that inhibits mTOR, reversed the increases in mTOR signaling and both prevented and treated the behavioral deficits. Only male mice were used, so sex effects could not be detected^63^.

In the three-chambered social approach test^38^, DISC1^D453G^ mice of either sex showed a preference for an unfamiliar mouse versus an empty container, and were clearly able to discriminate between the previously introduced mouse and a novel mouse. However, DISC1^D453G^ females spent less time than wild-type females engaged in social exploration of the unfamiliar mice. We found that the significant difference was largely driven by the wild-type females (rather than any other group) spending more time socially approaching the unfamiliar mice. Given that we used male mice as the novel social stimulus, it is possible that a form of sexual attraction is responsible for the increased social exploration by the wild-type females, which was blunted in the DISC1^D453G^ females. However, extensive validation using the same apparatus as currently employed found no detrimental effect of using male conspecifics as there were “no confounding sexual or aggressive behaviors, because the strangers were contained within the wire cages^64^”.

The DISC1^D453G^ mice that we tested had a predominantly C57BL/6N background but descend from an ENU-mutagenized BALB/cAnN male. On average, by backcross generation N_6_, 0.78% of their genome would have been of BALB/cAnN origin. However, the significantly lower open field activity shown by BALB/c mice compared with C57BL/6 mice^65^ suggests that the observed behavioral phenotype of DISC1^D453G^ mice is unlikely to reflect any residual contribution of the BALB/cAnN alleles surrounding *Disc1* at 125,054,195-125,261,858 bp on chromosome 8.

In summary, we have generated and analyzed mice carrying mutation D453G in order to probe the *in vivo* effects of missense mutation of DISC1’s C-terminal tail. Our initial analysis revealed that the behavioral effects of D453G were sex dependent. While female DISC1^D453G^ mice demonstrated a behavioral phenotype of novelty-induced hyperlocomotion, increased anxiety, and reduced social interaction of conspecifics, male DISC1^D453G^ mice displayed a deficit in passive avoidance. Consistent with the location of D453G within a predicted α-helical coiled-coil and a known GSK3β binding region, whole brain homogenates from DISC1^D453G^ mice of either sex showed evidence of interrupted GSK3β signaling, with decreased S9 phospho-inhibition of GSK3β, and decreased levels of β-catenin. Although D453G likely impacts binding to other proteins, our data suggest that interruption of GSK3β signaling may be part of the mechanism underlying the behavioral phenotype associated with D453G. This study thus adds to the converging evidence that GSK3β represents a pathogenic signaling pathway in schizophrenia and affective disorders.

## Methods

### Mutation Screen

Heteroduplex detection by high-resolution melting analysis in a LightScanner (Idaho Technology) was used to screen 7,776 male F_1_ progeny of ENU-mutagenized BALB/cAnN males and untreated C3H/HeH females in the MRC Harwell ENU DNA archive for mutations in exon 5 of *Disc1*, which was amplified using PCR primers F, 5′-TCT CCA CAC CTG TAC CAA TG-3′ and R, 5′-CCA CTC CCT TTC TGG AAG AT-3′. The A1358G mutation in *Disc1* was confirmed by DNA sequencing using a BigDye Terminator v3.1 Cycle Sequencing Kit (Applied Biosystems). To predict the potential effect of D453G on the function and structure of the DISC1 protein, the following three programs were utilized: PolyPhen-2 (genetics.bwh.harvard.edu/pph2)^29^, PMut (mmb.pcb.ub.es/PMut)^30^ and PROVEAN (provean.jcvi.org/seq_submit.php)^31^. The secondary structure within mouse DISC1 (NP_777279) was analyzed and annotated using the coiled-coil predictor COILS with a 21-residue window (ch.embnet.org/software/COILS_form.html)^46^ and the secondary structure predictor PsiPred (bioinf.cs.ucl.ac.uk/psipred)^47^. ClustalW2 (ebi.ac.uk/Tools/msa/clustalw2)^66^ was used to align the DISC1 protein sequences of mouse (ENSMUSP00000112410), sheep (ENSOARP00000003502), human (ENSP00000355593), soft-shelled turtle (ENSPSIP00000005750), green anole lizard (ENSACAP00000002671), chicken (ENSGALP00000040765) and zebrafish (ENSDARP00000131863). DISC1^D453G/+^ frozen sperm is available from the MRC Mammalian Genetics Unit, UK (har.mrc.ac.uk).

### Subjects

Heterozygous N_2_ backcross progeny of the founder DISC1^D453G/+^ (C3H/HeH × BALB/cAnN) F_1_ male (EMRCB/60.3d) and wild-type C57BL/6NTac females (Taconic) were backcrossed through the male and female lines to C57BL/6NCrl (Charles River) for four generations (N_3_-N_6_) before heterozygotes with a predominantly C57BL/6N genetic background (average of 98.4375% at N_6_) were intercrossed to generate homozygous DISC1^D435G/D453G^ mutants for phenotypic testing, which were viable and grossly indistinguishable from their wild-type DISC1^+/+^ littermates. Mice were weaned at 3 weeks of age and grouped housed (2–4 mice/cage) with same-sex littermates under a 12 hour light/dark cycle (lights on at 06.00). Pelleted feed (CRM-P, SDS) and water were provided *ad libitum*.

DNA was extracted from ear biopsies taken at weaning. Mice were genotyped for the DISC1^D453G^ mutation by the absence of a *Bse*NI restriction site (5′-ACTGGN*-3′) in a 411-bp fragment amplified using PCR primers F, 5′-AAG CCC CAA CAG ATC CTA GT-3′ and R, 5′-AAA GGA GTG GCC GCT CTA C-3′ with HotShot Diamond (Clent Life Science) using the thermocycling program of: 95 °C for 5 min, followed by 34 cycles of 94 °C for 30 s, 59 °C for 60 s, 72 °C for 40 s, followed by 72 °C for 10 min. PCR products were subsequently incubated with *Bse*NI (Fermentas) at 65 °C overnight and visualized by agarose gel electrophoresis, with a 253-bp band indicating the wild-type allele and a 411-bp band indicating the mutant allele. All procedures were approved by the University of Leeds Ethical Review Committee and conducted in accordance with the requirements of the Animals (Scientific Procedures) Act, 1986.

### Behavioral Experiments

All behavioral experiments were conducted using young adults over 8 weeks of age. Mice were handled for one week prior to behavioral testing. Mice were transferred to the experimental room 30 mins prior to the start of testing. All apparatus was cleaned with 70% ethanol between each mouse. Unless stated otherwise, all experiments were recorded using the tracking software ANY-Maze (Stoelting). Experiments were conducted in the order described below. A 2–3 day inter-test interval was employed for less severe experiments, and an inter-test interval of 1–2 weeks was used for the more invasive tests (PPI, passive avoidance). For all experiments, except PPI and passive avoidance (that were illuminated by LEDs within the chambers), the apparatus was illuminated by standard fluorescent ceiling lights at an intensity of ~200 lux. Experiments were conducted using identical apparatus and protocols as described elsewhere^67^. Methods are described briefly below.

### Open Field

Mice were placed into a 40 × 40 × 40 cm arena illuminated at ~200 lux at the center and were left to explore undisturbed for 30 mins. A webcam affixed to a tripod was positioned directly above the arena to record the trials. The tracking software divided the arena into the following zones, using the following dimensions: Outer Zone: 8 cm from the outer walls; Center Zone: 6.4 cm^2^ (16% of the total area); Intermediate Zone: the remaining area between the Outer Zone and the Center Zone. Time spent in each of the zones was measured, along with the distance travelled. The duration of rearing and grooming during the trial was measured by the experimenter.

### Elevated Plus-Maze

The elevated plus-maze consisted of two closed arms with white opaque walls (15 cm high) and two open arms. All arms measured 30 cm long and 5 cm wide. There was a central square measuring 5 cm^2^ where all arms met. The open arms were illuminated with an intensity of 200 lux, the central square with 150 lux, and the closed arms with 100 lux. Sessions commenced by placing the mouse onto the central zone facing the open arm opposite from the experimenter. Mice were allowed to explore the maze for 5 min and were then returned to the home cage. Mouse movement was tracked using ANY-Maze and time in the open arms, closed arms and central zone was measured, in addition to the frequency of head dips (downward movement of mouse’s head toward the floor from the open arms). Entry into an arm was defined as when the hind legs crossed the boundary of the arm.

### Forced Swim Test

A 5L glass beaker (17 × 27 cm) was filled with 3L of water at 25 ± 1 °C. The mouse was introduced into the water facing away from the experimenter and allowed to swim freely for 6 mins. Time spent floating was measured from 2 mins into the trial. Floating was defined as the lack of all movement except those required to keep the mouse afloat.

### Tail Suspension Test

The mouse was suspended by electrical tape attached near the tip of the tail, which was affixed to a structure that allowed the mouse to be suspended 20 cm from the ground. The trial lasted for 6 mins, during which the time spent immobile was measured. Immobility was defined as the lack of all movement except that required for respiration.

### Social Approach Test

Social approach was assessed using a three-chambered apparatus (60 × 40 cm), which had two doors to allow the mouse to access left and right chambers from a central compartment (each chamber being 40 × 20 cm). Following a 15-min habituation period, two cylindrical containers (10 × 10.5 cm, comprising vertical metal bars 9 mm apart) were placed into the left and right chambers, into one of which an unfamiliar adult male C57BL/6 mouse (age >10 weeks; ‘stranger 1’) was placed. Left/right placement was counterbalanced across groups. Time spent exploring each container was measured for 10 mins.

A second unfamiliar adult male C57BL/6 mouse (‘stranger 2’) was then placed into the empty cylinder and time spent exploring each container was measured for 10 mins. The time spent exploring stranger 1 or the empty cylinder in the first phase, and time spent exploring either stranger 1 or 2 in phase two were recorded. Due to a software recording error, only 9 male wild-type mice were included in the analysis of this experiment.

### Passive Avoidance

Passive avoidance was assessed using the Med Associates Shuttle Box controlled by MedPC software. The shuttle box consisted of two chambers, divided by a remotely operated guillotine door, and housed within a sound attenuation box with a fan generating background noise. One half of the chamber was darkened using a black cloth cover (defined as the ‘dark chamber’). The other side of the chamber was illuminated by a light bulb. During the conditioning phase, latency to cross from the light chamber to the dark chamber was measured. When the mouse fully entered the dark chamber, the door closed and, 10 s later, a 0.45 mA footshock lasting 3 s was delivered. The mouse was removed 30 s after the footshock. 24 hours later, the latency to cross to the dark chamber was measured in a probe trial. The prove trial was stopped when either the mouse traversed to the dark chamber or if had not done so within 300 s.

### Prepulse Inhibition

Mice were tested for acoustic startle reactivity (ASR) and prepulse inhibition (PPI) using the SR-LAB startle response system (San Diego Instruments), which consists of a sound attenuating box housing an animal enclosure platform, a fan and a speaker (all of which were lit from above by LEDs). Acoustic startle responses were measured at 70 dB, 80 dB, 85 dB, 90 dB and 120 dB. PPI was measured by the delivery of a tone at either 70 dB, 80 dB, 85 dB or 90 dB (prepulse) for 10 ms followed by a 100 ms gap with background noise and then a 120 dB ‘startle’ tone for 40 ms. Background noise at 65 dB was presented throughout the trials. 10 presentations of each ASR and PPI tone were given and the responses averaged. Inter-trial intervals averaged 25 s.

### Nesting Behavior

This test was based on a published protocol^68^. Mice were individually housed before the onset of the dark phase of the light/dark cycle with no environmental enrichment apart from a 3 g ‘nestlet’ (Lillico) of pressed cotton. Nesting ability was assessed the following morning at 0800 h and was rated using the following scale:

1. Nestlet not noticeably touched (>90% intact); 2. Nestlet partially torn up (50–90% intact); 3. Nestlet mostly shredded but no identifiable nest site; 4. Identifiable nest, but flat; 5. A perfect or near-perfect nest.

## Protein Analysis

### Tissue Lysates

Using a dounce homogenizer, single hemispheres were homogenised in lysis buffer consisting of 20 mM Tris-HCl (pH 7.5), 150 mM NaCl, 1 mM Na_2_EDTA, 1 mM EGTA, 1% Triton, 2.5 mM sodium pyrophosphate, 1 mM β-glycerophosphate, 1 mM Na_3_VO_4_, 1 μg/ml leupeptin, and protease and phosphatase inhibitors (Roche), and incubated at 4 °C with constant agitation for 90 mins. Lysates were cleared by centrifugation at 14,000 rpm for 20 mins (4 °C) and protein concentration was determined using the Bradford assay. Protein concentration was equalized in all samples.

### Antibodies

The following primary antibodies were used: anti-DISC-1 (1 in 1000, sc-47990, Santa Cruz), anti-GSK3β (1 in 1000, sc-9166, Santa Cruz), anti-phospho-GSK3β (Ser9) (1 in 1000, 9322, Cell Signaling), anti-β-catenin (1 in 1000, ab16051, Abcam), and anti-GAPDH (1 in 10,000, ab8245, Abcam).

### Western Blotting

Samples were prepared by boiling in Laemmli buffer (10% SDS, 300 mM Tris-HCl, pH 7.2, 0.05% bromophenol blue, 50% glycerol, 10% β-mercaptoethanol) at 95 °C for 5 min, and resolved using the NuPAGE system with 4–12% pre-cast gels (Life Technologies) alongside protein standards (Precision Plus, BioRad). Proteins were then transferred onto nitrocellulose membranes (Amersham) at 30 V for 75 mins. The membranes were then blocked in 5% (w/v) Bovine Serum Albumin (BSA) in TBS-T (25 mM Tris-HCl; pH 7.6, 100 mM NaCl, 0.5% Tween-20) for 1 hour at room temperature. Primary antibodies were diluted in 1% (w/v) BSA in TBS-T, and blots were incubated overnight at 4 °C. Bound antibody was detected using fluorescently conjugated donkey anti-goat, anti-rabbit or anti-mouse secondary antibodies (1:10,000, Licor Biosciences) and visualized using the Licor Odyssey Imaging system (Licor Biosciences). Quantification of the band intensity was accomplished by densitometry using Image Studio Lite v.5 (Licor Biosciences). All experiments were run in triplicate.

### Immunoprecipitation

For immunoprecipitation, a DISC1 antibody was used to immunoprecipitate endogenous GSK3β. The resulting immunocomplexes were captured using Protein G beads (GE Healthcare) and incubated on a rotating wheel overnight (4 °C). The immunocomplexes were then collected by centrifugation at 10,000 × g for 5 min and washed three times with lysis buffer. Isotype-matched IgG (Sigma-Aldrich) was used as a negative control to screen for non-specific binding. Bound proteins were then eluted in Laemmli sample buffer and subjected to Western blotting as described above.

### Data Analysis

All data are expressed as mean ± standard error of the mean (SEM). All datasets were initially checked for normality and homogeneity of variance. To assess differences between the variables and their impact upon performance, analysis of variance (ANOVA) was conducted, with the factors of genotype and sex always examined. Performance across time bins or days was analyzed by repeated measures ANOVA. If there were significant interactions between variables, tests of simple main effects (SME) were performed (Bonferroni corrected for multiple comparisons), followed by *post hoc* analysis when necessary. The ranking of the nest building was transformed according to the mean scale, and a two-way ANOVA was performed. All analyses were performed using SPSS (version 20). In all cases, α was set at < 0.05. Graphs were prepared using GraphPad Prism version 6.

## Additional Information

**How to cite this article**: Dachtler, J. *et al.* Missense mutation in DISC1 C-terminal coiled-coil has GSK3β signaling and sex-dependent behavioral effects in mice. *Sci. Rep.* **6**, 18748; doi: 10.1038/srep18748 (2016).

## Supplementary Material

Supplementary Information

## Figures and Tables

**Table 1:** Summary of observed phenotypes of DISC1^D453G^ mutant mice.

Phenotype	Male DISC1^D453G^	Female DISC1^D453G^
Open Field		
Distance travelled (m)	=	↑
Time spent in outer zone	=	↑
Time spent in center zone	=	↓
Rearing (s)	↓	=
Elevated Plus-Maze		
Time spent in open arms (%)	=	↓
Open arm entries	=	↓
Time spent in closed arms (%)	=	↑
Time spent in center (%)	↑	=
Head dips	=	↓
Social Interaction		
Novel mouse vs. empty container	=	↓
Stranger 2 vs. Stranger 1	=	↓
Passive Avoidance		
Latency to cross (24 hours)	↓	=
DISC1-GSK3β pathway		
DISC1 protein level	=	=
DISC1-GSK3β binding	↓	↓
GSK3β protein level	=	=
Phospho-inhibition of GSK3β	↓	↓
β-catenin protein level	↓	↓

= no significant difference, ↓ decreased, ↑ increased; versus wild-type of the same sex.
