# Supplementary material for: Missense mutation in DISC1 C-terminal coiled-coil has GSK3β signaling and sex-dependent behavioral effects in mice
Source: Sci Rep. 2016 Jan 5;6:18748. doi: 10.1038/srep18748 (PMC4700527; doi:10.1038/srep18748)
Supplement: Supplementary Information [file srep18748-s1.pdf]

Supplementary Information

Missense mutation in DISC1 C-terminal coiled-coil has GSK3β signaling and sex-dependent behavioral effects in mice

James Dachtler, Christina Elliott, R. John Rodgers, George S. Baillie & Steven J. Clapcote

Supplementary Fig. S1

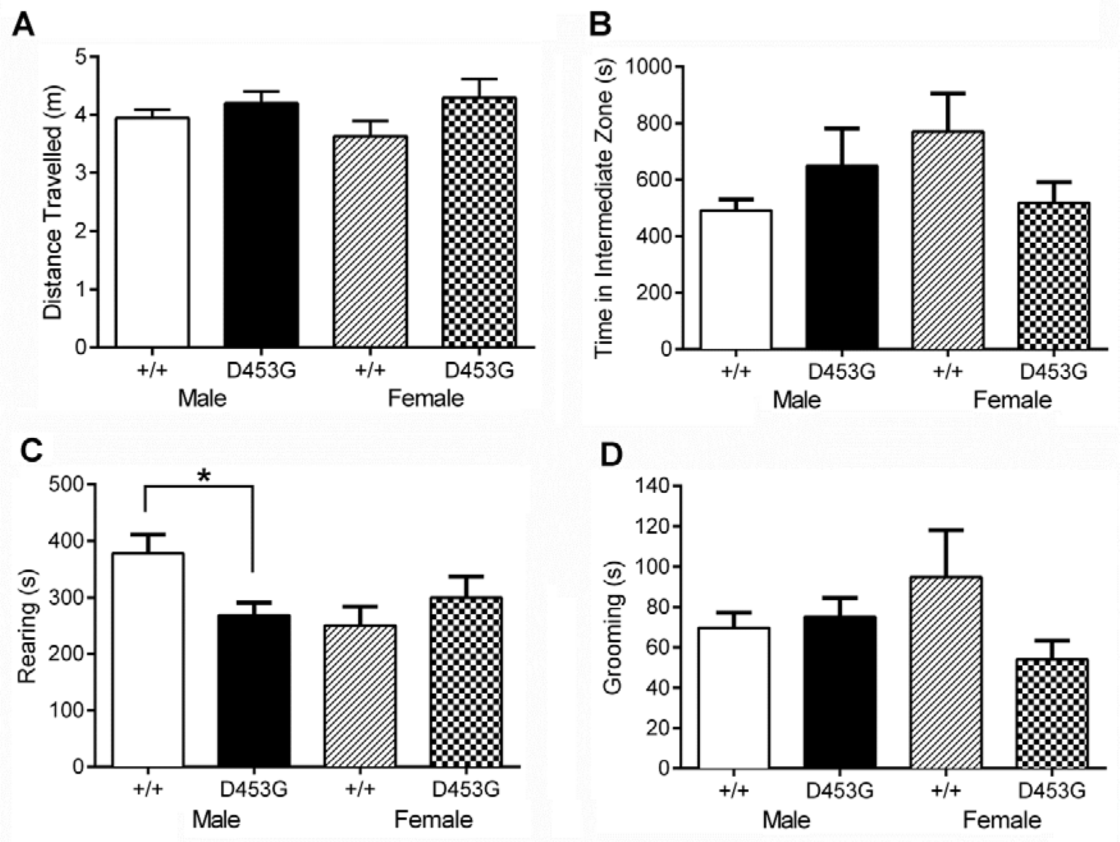

Supplementary Figure S1. Measures of exploration in the open field. (**A**) Distance travelled during the first min in the open field showed no significant differences between genotypes or sexes. (**B**) The floor of the open field arena was split into three zones: outer, intermediate and center. There was a difference approaching significance between female wild-type (+/+) and DISC1^{D453G} (D453G) mice ($P = 0.062$) for the time spent in the intermediate zone. (**C**) Male DISC1^{D453G} mice were found to spend less time making exploratory rearing movements than wild-type males. (**D**) All groups spent a similar time self-grooming. *$P < 0.05$ vs. male +/+.

Supplementary Fig. S2

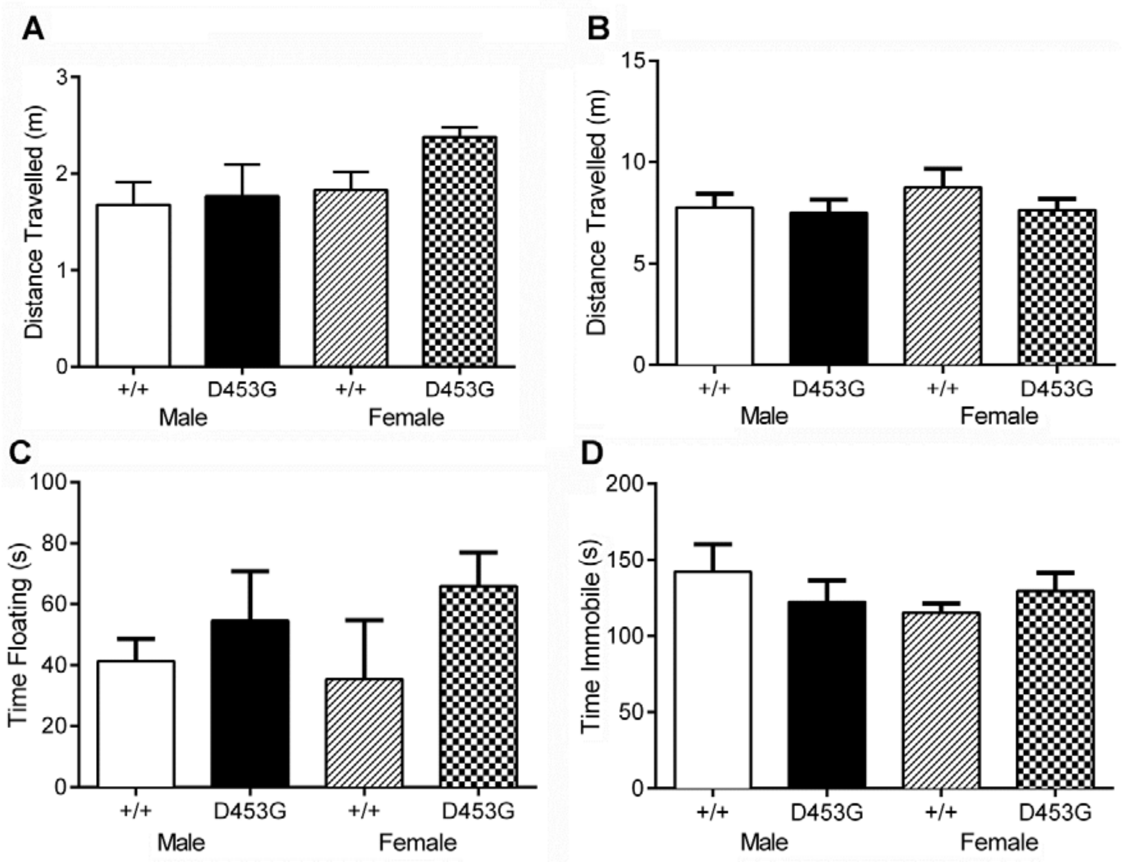

Supplementary Figure S2. Behaviors in the elevated plus-maze, forced swim test and tail suspension test. Total distances travelled during the first min (**A**) and throughout the 5 min elevated plus-maze test (**B**) were not significantly different between genotypes or sexes. (**C**) No significant effect of DISC1^{D453G} on the duration of floating in the forced swim test. (**D**) No significant effect of DISC1^{D453G} on the duration of immobility in the tail suspension test.

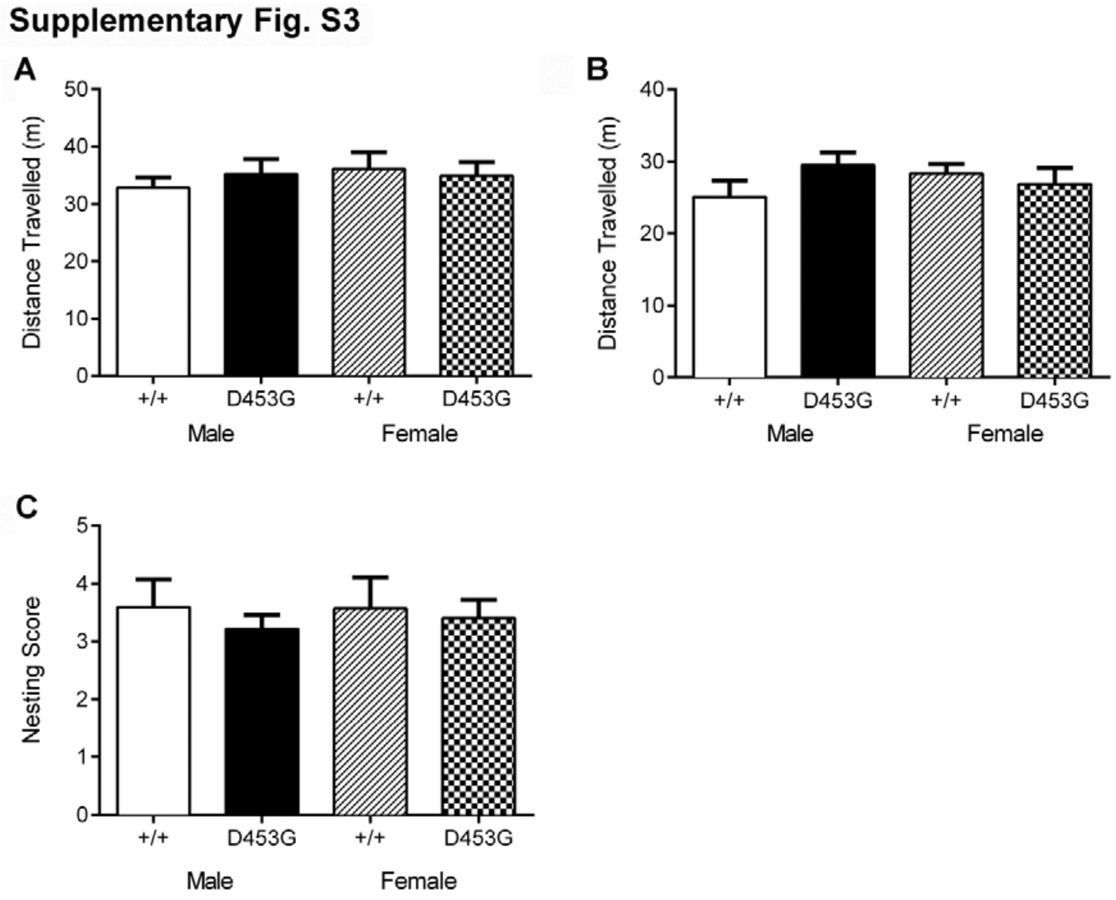

Supplementary Figure S3. Social approach test ambulation and nesting score. The total distance travelled during the three-chambered social approach test was similar between all groups in (**A**) phase 1 (sociability: stranger 1 vs. empty container) and (**B**) phase 2 (social recognition: stranger 2 vs. stranger 1). (**C**) No significant genotypic differences in nesting ability, rated on a scale of 1-5. +/+: wild-type; D453G: DISC1^{D453G}.

Supplementary Fig. S4

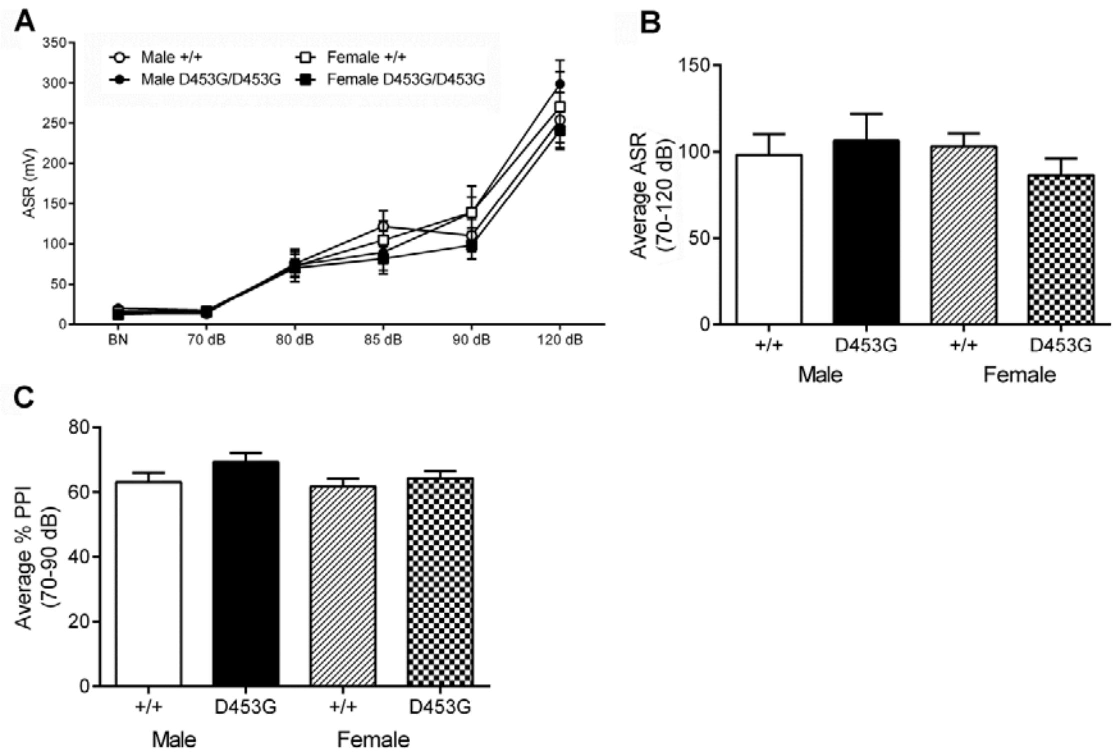

Supplementary Figure S4. Acoustic startle reactivity and average PPI. (**A**) Tones of varying intensities (70-120 dB) were presented to mice and the startle response was measured. No genotypic differences in startle response magnitude were observed at any of the tone intensities. (**B**) Acoustic startle reactivity to varying tones (70-120 dB) was averaged. No significant differences in average ASR were observed between the genotypes or sexes (two-way ANOVA, genotype and sex ($F_{(1, 30)} < 1$), sex x genotype ($F_{(1, 30)} = 1.14$, $P > 0.05$)). (**C**) Prepulse inhibition to all of the prepulse tones (70, 80, 85 and 90 dB) were averaged. No significant differences in PPI were observed between the genotypes or sexes (two-way ANOVA, genotype ($F_{(1, 30)} = 2.68$, $P > 0.05$), sex

($F_{(1, 30)}$ = 1.46, P > 0.05), sex x genotype ($F_{(1, 30)}$ < 1, P > 0.05)). +/+: wild-type; D453G: $DISC1^{D453G}$.
